# Fabrication of millet starch nanocapsules loaded with beta carotene using acid hydrolysis and ultrasonication: Characterisation, release behaviour and bioactivity retention

**DOI:** 10.1016/j.ultsonch.2024.107112

**Published:** 2024-10-16

**Authors:** Mehak Nazir, Faiza Jhan, Asir Gani, Adil Gani

**Affiliations:** a Department of Food Science and Technology, University of Kashmir, Srinagar 190006, India; b School of Bioengineering and Food Technology, Shoolni University, Solan, Himachal Pradesh 173229, India

**Keywords:** Starch, Nanoencapsulation, Ultrasonication, Beta carotene, Bioactivity

## Abstract

The acid hydrolysis process was used to create novel millet starch-based nanoparticles from three different sources: sorghum, foxtail millet and pearl millet. An environment-friendly, risk-free ultrasonication technique was used for encapsulating beta carotene in starch nanoparticles to create nanocapsules that will shield the bioactivity of beta carotene in gastrointestinal conditions and increase its accessibility after consumption. Formulated nanocapsules were examined for zeta potential, particle size and encapsulation efficiency. The particle dimensions of beta carotene-loaded sorghum (SSB), foxtail millet (FSB), and pearl millet (PSB) starch nanoparticles were 416, 399 and 587 nm with zeta potential of −17.98, −19.03 and –22.31 mV respectively. Encapsulation efficiencies of nanocapsules were found to be 85.83, 89.65 and 78.32 % for SSB, FSB and PSB respectively. Scanning electron microscopy (SEM) was also harnessed as a confirmatory tests towards the presence of beta carotene in nanocapsules. Beta carotene encapsulation in starch nanoparticles was also demonstrated using ATR-FTIR which revealed broad characteristic peaks at 3000, 1086 and 885 cm^−1^ that occur without any discernible interaction. Intestinal juice with higher beta carotene content ensured controlled release in the intestine. Encapsulated beta carotene showed more bioactive properties in terms of antioxidant activity as compared to free beta carotene form.

## 1 Introduction

Starch is a sustainable, organic and environmentally benign material that a lot of plants produce to store energy. Starch carbohydrates are predictably the predominant polymers in the environment. It has a variety of uses as a core material in various sectors of the food, pharmaceuticals and textile industries because of properties including biocompatibility, shelf stability, low levels of toxicity and excellent drug delivery vehicle [1]. Wheat, maize, cassava, buckwheat, millet and potatoes are the principal sources of native starch. Worldwide millets are recognized as a valuable grain, yet their full potential remains largely untapped. Millet grains boast abundant nutrients and health-promoting phenolic compounds, rendering them suitable for both human consumption and animal feed [2]. Studies indicate that millets exhibit strong antioxidant activity and contribute for promoting better health [3]. Starch makes up a significant portion of millet grains roughly 70 % of the grain overall. Starch present in millets consisits of 20–30 % of amylose and remaining 70–80 % is made up of amylopectin [4]. Key characteristics of millet starch include its ability to swell, viscosity, digestibility, degree of gelatinization, structural qualities and hypoglycaemic properties compared to other cereal starch [5]. Its industrial uses in the food, packaging and medical sectors are influenced by these attributes both directly and indirectly. Although, there are major constraints to this polymer that hinder its widespread usage in the nutritional food business. The drawbacks involve the polymer’s propensity to retrograde, extreme sensitivity to temperature and humidity, inability to dissolve in cold water and resistance against enzyme hydrolysis. They typically demonstrate a retrogradation tendency when it comes to pH, temperature and shear pressure variations [6]. These limitations are circumvented by making a few modifications. An assortment of physical, chemical, and enzymatic alterations can enhance the functional characteristics of starch rendering it more versatile for a wide range of uses [7]. This indicates that starch frequently needs to be altered to improve its qualities. The nano-reduction of this macromolecule is one such modification, which is popular right now because of its tailored small stature, enhanced functional features, higher physical resilience, better bioactive persistence, enhanced and precisely expulsion behaviour of encapsulated functional components [8]. Through several methods, starch may be utilized to build nanostructures that result in regeneration and precipitation, producing particles with various properties, crystal structures and morphologies [9]. For the manufacture of starch nanoparticles, there are numerous methods available such as irradiation, ultrasound, breakdown of starch through acid, enzymes, or a combination thereof which can be described as hydrolysis. The acid hydrolysis in this instance is an efficient method of nanoreduction. Acid concentration, category, and breakdown duration may affect the microscopic structure and functional characteristics of starch. These starch variants are then employed across various applications including food, paper, textiles, and other commodities [10]. The physical characteristics and protective abilities of sustainable nanocomposites are enhanced by using appealing starch nanoparticles produced by acid hydrolysis [11], [12]. Additionally, the hydrolysis using acids followed by amylolysis may dramatically decrease the digestibility of starch. [13].The hydrolysis kinetics demonstrates the two steps of the procedure. The initial quick step destroys the unstructured layers within the starch granule, whereas the second slower phase degrades the crystallites [14], [15]. The exteriors of the granules which contain both amylose and amylopectin, are concurrently attacked in the early stages of acid hydrolysis resulting into the change in morphology and size of starch. Moreover, numerous sensitive food components have been effectively encapsulated using starch with high encapsulation efficiencies [16]. The purpose of encapsulation is to protect, extend or maintain the internalized substance from degradation in surroundings, thus allowing drugs as well as nutrients to be delivered more efficiently in biological systems [17]. Furthermore, the nano starch has large surface area-to-volume ratio and biological suitability, which can be used to encapsulate bioactive chemicals for culinary and pharmaceutical purposes. Ultrasonication is a versatile technique that has demonstrated success in the fabrication of functional compounds utilized across various sectors like food industries, image analysis, energy creation and curative/diagnostic medicine [18]. Acoustic cavitation, or the production and bursting of air bubbles caused by ultrasound, is the main mechanism responsible for nanomaterial formation [19]. Ultrasound can be utilized to replace or modify traditional procedures for creating encapsulating particles in aqueous environments, such as emulsification and polymerization [20]. Beta-carotene is a vital carotenoid for the well-being of people. It is generally acknowledged that beta-carotene has twice the vitamin A activity of alpha-carotene or beta-cryptoxanthin. But its poor natural absorption has prompted the emergence of encapsulation techniques like ultrasonication to increase stability and accessibility [21].

The starch in this study was isolated from millets viz., sorghum, foxtail millet and pearl millet. Their nano reduction was accomplished by the use of acid hydrolysis, and beta carotene encapsulation was achieved through ultrasonication, which is a unique, safe, and environmentally friendly technology. The study’s goal was to investigate the structural characteristics, release behavior and neutraceutical potentials of formulated nanocapsules.

## 2 Materials and methods

### 2.1 Materials

The chemicals and reagents used for the current study were of certified analytical grade. Beta carotene, pepsin, pancreatic enzyme, bile salts, phosphate buffer saline, DPPH (1,1−diphenyl−2−picrylhydrazyl), sodium phosphate buffer, aqueous potassium ferricyanide, ferric chloride, trichloroacetic acid and sodium carbonate were some of the important chemicals used in this research and were bought from Sigma Aldrich, St. Louis, USA. Millets such as sorghum, foxtail millet, and pearl millet were purchased from J&K’s regional markets.

### 2.2 Flour Preparation

To prepare the flour, cereal grains were first conditioned by soaking in water for whole night at 30 °C. Afterward, they were dried overnight at 40 °C in a hot air oven (NSW-143; Narang Scientific works Pvt. Ltd., New Delhi, India) until a final moisture content of 12–14 % was reached. Seeds were then pulverized using a mixer grinder (Sujata, New Delhi, India) and flour obtained was sifted through a 60-mesh sieve (BS Standard). The resulting whole grain flours were then kept at room temperature in airtight containers for future use.

### 2.3 Starch extraction

Flour was steeped in an alkaline solution to extract starch [22]. Flour was diluted with ultrapure water to a maximum of ten parts water to one part flour (w/v) to create a liquid slurry, the pH was kept at 9.5 by adding NaOH. Slurry was filtered through a muslin cloth. The filtrate was then centrifuged (5810, Eppendorf, Hamburg, Germany) at 1811 × *g* for 15 min, the supernatant was removed by decanting, leaving the pellet behind. The impurity-containing top film of pellet was scraped away to remove the sludge. The sediment was reintroduced in double distilled water before being centrifuged/ whirled once more. The same washing procedure was done three times to eliminate any impurity left. The starch extracted was dehydrated at 40°C in a hot air oven for one hour and then stored.

### 2.4 Acid hydrolysis of starch

The hydrolysis was carried out using the technique as described by Shah et al. 2017 [23]. Briefly, native starches were suspended (10 g dry starch per 250 mL) in 2 M HcL. The sample mixtures were shaken lightly regularly to re-suspend the accumulated granules. The storage vessel was tightly covered by a cap then stored at ambient temperature (20 °C) for 13 days. After that, samples were collected and centrifuged at 3220 × *g* for 15 min. The residue, or remaining material, was dried overnight at 40 °C under an air stream after being washed with distilled water as long as the clarified extracts achieved a neutral pH.

### 2.5 Ultrasound-assisted beta carotene loaded starch nanoparticles

Beta carotene (2 g) was introduced into a 40 ml starch solution and treated with ultrasonication using a Cole-Parmer instrument (model 04711–35, Mumbai, India) equipped with a 12 mm diameter probe dipped into the sample at 10 mm [24]. The sonicator was operating at 220 V in a pulse mode of 3 s on and 1 s off, for a duration of 30 min. Subsequently, the sonicated mixture underwent centrifugation at 3220 × *g* for 10 min. The resultant starch particles enriched with beta carotene, was harvested and subjected to oven drying at 30 °C for 12 h. Following drying, the sample was finely pulverized using a mortar and pestle.

### 2.6 Measurement of particle size and zeta potential

The characteristic particle size and zeta potential of nano-reduced starch particles were investigated using a particle analyzer (Litesizer, 500, Anton Paar, Austria). Sample (0.01 % w/v) was dissolved in deionized water (Elix −10, Millipore, Molsheim, France) and sonicated in the bath sonicator (Jain sonicator, India) at a frequency of 40 KHz for 20 min. The measurements were done at neutral pH and 25 °C.

### 2.7 Encapsulation efficiency

Following the approach of Shabana et al., 2018 [25], the encapsulation efficiency of starch nanoparticles for beta-carotene was determined. Briefly, samples containing beta carotene loaded starch nanoparticles (20 mg) were centrifuged at 1811 × *g* for 5 min. To release the trapped beta-carotene from the nano-encapsulated samples, the pellet was reconstituted in double-distilled water (2.5 mL) and sonicated for 20 min using a bath-type and then centrifuged one more time. The resulting clear liquid was filtered through whatman no. 1 and its absorbance was measured at 440 nm with a UV–Vis spectrophotometer. The beta-carotene content was assessed by measuring the absorbance at 440 nm of a known quantity of beta-carotene using a calculation based on a standard curve. The encapsulation efficiency (EE) of starch nanoparticles was then estimated harnessing the following formulas;

$$EE\left(\%\right)\;of\;starch\;nanoparticles=\mathrm{Beta}-carotene\;released\;from\;wall\;material/\mathrm{Total}\;beta-carotene\;added\;initially\times 100$$

### 2.8 Scanning electron microscopy of nanocapsules

The morphological investigation of nanocapsules was carried out in vacuum using a FE-SEM (GeminiSEM 500 8203017193, UK/GB). The samples were placed on a sticky strip that was adhered to a gold-covered, aluminum sample mount. At a 20 kV accelerator voltage, the samples were captured with a digital image analyzer.

### 2.9 Structure analysis by ATR-FTIR Spectroscopy

Utilizing FTIR spectrometer equipment (Cary 630 FTIR, Agilent Technologies, USA), the spectrum characteristics of the nanocapsules were assessed. The acquired spectra varied between 40 and 4000 cm^−1^ at an accuracy of 4 cm^−1^.

### 2.10 Release behaviour under simulated gastrointestinal conditions

The behaviour of beta-carotene release from nanocapsules was studied using simulated gastric and intestinal digestion [26]. The digestive fluid was made by combining gastric protease/ pepsin (3 g/L) with sodium chloride solution (9 g/L) and adjusting the pH to 3 with HCL (1 mol/L). The bile salts (3 g/L) and pancreatic digestive enzyme (10 g/L) were mixed in phosphate buffer saline (pH 7.5) to make the intestinal juice. The sample (100 mg) was placed in a vial with 10 mL of gastric juice, then it was allowed to incubate at 37 °C with gentle stirring. After 1.5 h of incubation, aliquots of 2 mL were collected. The remaining solution was centrifuged for 5 min at 1811 × g and the residue was recovered. This residue was incubated again at 37 °C with 10 mL of intestinal juice. The samples were gently swirled throughout incubation. After 3 h of incubation, an aliquot (2 mL) was collected. The supernatant was recovered after centrifuging all of the aliquots at 3220 × g for 5 min. The beta-carotene content of aliquots was ascertained through the measurement of absorbance at 440 nm using a UV–Vis spectrophometer (U-2900, Hitachi, Tokyo, Japan).

### 2.11 Sample preparation for anti-oxidant activity

The sample concentrations (w/v) were obtained by dissolving 20 mg of starch in 1 mL of ultrapure water and then sonicating for 20 min to guarantee proper mingling of mixture. The resultant mixture was then used to determine several antioxidant tests.

#### 2.11.1 DPPH radical scavenging activity

The sample’s DPPH (1,1-diphenyl-2-picrylhydrazyl)* scavenging capacity was measured using a modified technique published by Ahmad et al., 2017 [27]. A 20 mg/mL sample (5 mL) was mixed with a 5 mL methanol solution containing 60 mM DPPH. The product was swirled for nearly a minute before being stored at 25 °C in a dark room for 35 min. The absorbing capacity of the resulting combination was measured at 517 nm against a blank with a UV–vis spectrophotometer (U-2900, Hitachi, Tokyo, Japan). Methanol, dissolved in double distilled water, served as a positive control. The equation below was used to compute DPPH * scavenging ability.

$$\mathrm{DPPH}*scavenging\;activity\left(\%\right)=\left(1-A_{s}/A_{c}\right)\times 100$$

Where “A_c_” is the absorbance of the control, “A_s_” is the absorbance of the sample.

#### 2.11.2 Metal chelating activity

At room temperature, the absorption capacity of the resulting combination was evaluated against an untreated control at 562 nm and compared to citric acid (standard) [1]. The ferrous ion chelation capacity of each sample was calculated as a percentage chelating impact utilizing the following formulas:

$$\mathrm{Chelating}\;effect\left(\%\right)=A_{c}-A_{s}/A_{c}\times 100$$

Where “A_c_” is the absorbance of the control and “A_s_” is the absorbance of the sample.

#### 2.11.3 Reducing power

The reducing power of extracts was determined using the method described by Wani et al., 2017 [28]. Different amounts of starch working solution (1 mL) were blended with 2.5 mL of sodium phosphate buffer (0.2 M, pH 6.6) and 2.5 mL of aqueous potassium ferricyanide (1 % w/v) and then vortexed. This mixture was incubated at 50 °C for 30 min. After that, 2.5 mL of 10 % w/v trichloroacetic acid was added to the incubated mixture, followed by 10 min of centrifugation at 1811 × g. Supernatant (2.5 mL) was extracted from this and diluted with distilled water (2.5 mL) and ferric chloride (0.5 mL, 1 % w/v). The absorbance of mixtures was measured at 700 nm in comparison to a blank. Percent reducing power was calculated from the formula:

$$\text{Re}ducing\;power\left(\%\right)=\mathrm{Absorbance}\;of\;the\;sample/\mathrm{Absorbance}\;of\;the\;control-1\times 100$$

### 2.12 Statistical analysis

To calculate the statistical analysis of findings (mean values, standard deviation, ANOVA), the commercial statistical tool SPSS (IBM Statistics 22) was utilized. Duncan’s tests were used to evaluate the data at a 5 % confidence level.

## 3 Results and discussion

### 3.1 Particle size

Table 1 presents the particle size measurement of beta-carotene loaded starch nanocapsules. The hydrodynamic particle size of the nanocapsules, namely SSB, FSB, and PSB, was observed to be 416, 399, and 587 nm, respectively. The reduction in particle size can be attributed to the breakage or degradation of starch granules induced by acid hydrolysis and the ultrasonication process. The impact of ultrasonic frequencies, which generate cavitation and the bursting of microbubbles, facilitates particle degradation, fragmentation, and chemical rearrangement, thereby achieving nanoscale particle size for all encapsulated particles [29].

**Table 1:** Particle size, zeta potential, and encapsulation efficiency of nanocapsules.

Sample	Particle size (nm)	Zeta potential	E. E
SSB	416.32	−17.98	85.83 ± 0.02^b^
FSB	399.21	−19.03	89.65 ± 0.12^c^
PSB	587.98	–22.31	78.32 ± 0.32^a^

Where SSB, FSB, and PSB represent Beta carotene loaded starch nanocapsules prepared from sorghum, foxtail millet, and pearl millet starch respectively. The presence of distinct small superscript letters (a, b & c) on the mean data alongside the standard deviation (±) within the identical column indicates significant differences (*p* < 0.05) among them.

### 3.2 Zeta potential

Zeta potential determines colloidal stability and average electric potential on the particles. SSB (−17.98 mV), FSB (−19.03 mV), and PSB (–22.31 mV) particles all had an absolute higher negative charge after nano-reduction. Similar results were also found by Ahmad et al., 2019 [30]. The negative potential might be due to the degradation of molecular bonding and accessibility to a negative surface electric field. This surface charge also determines the stability of dispersions. The stability increases as the negative zeta potential increases because there are more electrostatic repelling forces among the particles and fewer van der waal forces, which stops the particles to aggregate [31]. Sample PSB showed more stability than FSB and SSB due to their greater zeta potential. The total charge on particles was negative, which might be attributable to the existence of organic phosphate ester ions in amylopectin molecules as well as the negative charge of beta carotene [30].

### 3.3 Encapsulation efficiency

One of the most crucial quality factors that affect a wall material’s capacity to enclose or support the core material inside is encapsulation efficiency. Table 1 displays the encapsulation efficiency findings of our study. The encapsulation efficiency, which is defined by the number of drugs initially loaded during the encapsulation process, expresses the proportion of the primary substance trapped inside the protective coating. In this study, the percent beta-carotene nanoencapsulated in millet starch nanoparticles was found to be 85.83 (SSB), 89.65 (FSB), and 78.32 % (PSB), respectively. The findings suggest that millet starch nanoparticles can entrap beta carotene in its core efficiently. This might be due to starch’s film-forming capabilities, the core-like structure may be primarily generated [32]. Low encapsulation efficiency for drugs with high loading percentages is explained by the probability that the high ratio of active material cannot completely entrap it. As a result of the low initial drug load in our study, beta-carotene was effectively encapsulated in starch nanoparticles. FSB and SSB starch nanoparticles demonstrated noticeably better encapsulation efficiencies than PSB starch among the three sources of starch. These results indicate that starch granules with lowest size among all samples (Table 1) showed better encapsulating efficiency [26].

### 3.4 Scanning electron microscopy

**Fig. 1:**
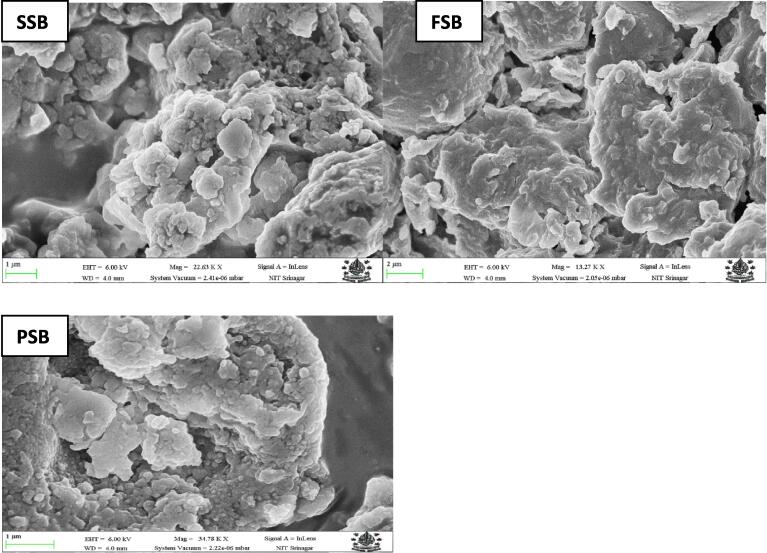
Scanning electron micrographs of SSB, FSB, and PSB represent Beta carotene nanocapsules prepared from sorghum, foxtail millet, and pearl millet starch, respectively**.**

The exterior morphological structure of nano-encapsulated beta carotene particles was observed by using SEM displayed in Fig. 1. The granular form of starch from sorghum, foxtail millet, and pearl millet was similar in the observation as shown in Jhan et al., 2020 [1]. Shape variations were noted in all three samples. The structure seems uneven, rough, inhomogeneous, and porous in the SSB micrographs, which are excellent for encapsulating the beta-carotene inside the cavities. Additionally, FSB has several channels and pores and displays a paste-like structure with some embedded beta-carotene particles. PSB has a rough, irregular structure with pores and crevices that are also filled with beta-carotene. The ultrasonic cavitation process disorganized the particles owing to turbulence, shock, and shear stresses, reducing particle size and exposing cleave bonds that retain the bioactive substances more effectively [31]. In our analysis, fragmentation was the only discernible change; samples that had undergone ultrasound treatment still had most of the starch surface features. The trapped acoustic energy by granules of starch produces vibrations with high frequencies that rupture the starch network. The SEM investigation revealed that beta carotene was encapsulated within the spaces, pores, or sheets created by starch nanoparticles.

### 3.5 Attenuated total reflectance-fourier transform infrared spectroscopy (ATR-FTIR)

**Fig. 2:**
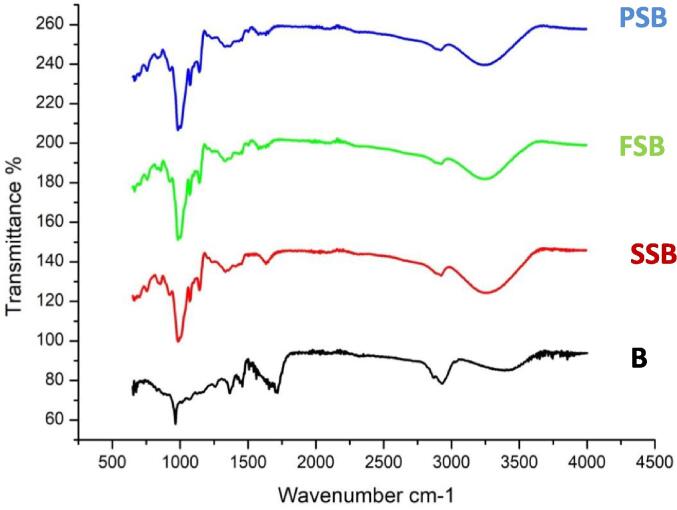
FTIR analysis of Beta carotene (B), Beta carotene loaded sorghum starch (SSB), foxtail millet (FSB) and pearl millet (PSB) nanocapsules, respectively.

ATR-FTIR analysis identifies the molecular spectra of chemical compounds, which is especially helpful for characterizing and analysing molecular interactions in micro- or nanoparticles. Fig. 2 depicts the molecular structure of starch nanoparticles with encapsulated beta carotene. Starch’s unique bands are generated by vibrations at amylopectin and amylose units. All samples showed peak absorption levels in the 3200–3500 cm^−1^ region, which corresponds to the expansion of O −H bonds in starch. Another peak of absorption was found in the 2925–3000 cm^−1^ range, which is attributed to vibration generated by the elongation of C−H bonds in the starch glucose unit. Whereas the distinctive peaks around 1645 cm ^- 1^ are because of the existence of water that is closely bound in starch, the absorbance bands around 1410 cm ^- 1^ and 1148 cm ^- 1^ indicate the widening of anhydrous glucose ring C−H and C−O−C in starch samples, peaks around 993 cm ^- 1^ are due to stretching of C −O and C−O−C. [26]. Many additional functional groups of beta carotene, such as the CH group stretched between wavelengths 2900 and 3000 cm^−1^ and the aromatic C = C group extending at 1620, 1086 and 885 cm^−1^ were also present in the aliquot of nano-encapsulated beta carotene solution. [21]. The existence of these distinctive peak patterns in an aliquot of nano encapsulated beta carotene suspension highlights the encapsulation of beta carotene inside the nanostructure of starch nanoparticles.

### 3.6 Release behaviour of beta carotene from nanocapsules

**Fig. 3:**
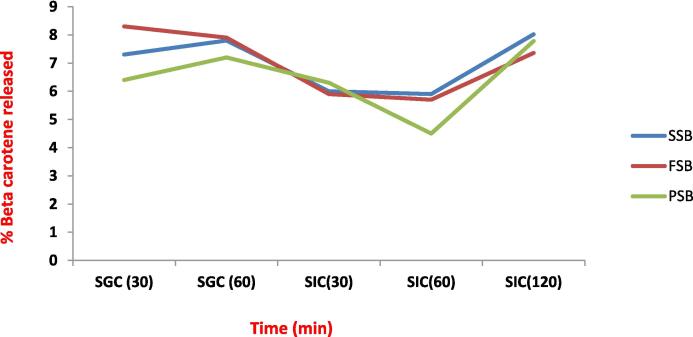
SSB, FSB, and PSB represent beta carotene nanocapsules prepared from sorghum, foxtail millet and pearl millet starch respectively. SGC and SIC represent simulated gastric conditions (60 min) and simulated intestinal circumstances (120 min), respectively.

The primary goal of nanoencapsulation was to shield the bioactive molecule i.e., beta carotene from the severe environment present in the gastrointestinal system and allow it to be released at the targeted spot to maintain the intended health benefits. Fig. 2 depicts the *in vitro* release behaviour of beta-carotene encapsulated in millet based nano-starch structures under simulated stomach and intestinal circumstances. Under simulated gastric conditions, SSB, FSB, and PSB released 7.3 µg/mL, 8.3 µg/mL, and 6.4 µg/mL of beta carotene during the initial 30 min of digestion. The following 60 min revealed little substantial variations among the SSB, FSB, and PSB, which released 7.8 g/mL, 7.9 g/mL, and 7.2 g/mL beta carotene, respectively. Thus, when exposed to simulated gastric conditions, PSB had the greatest delayed release, followed by SSB and FSB. The existence of non-encapsulated beta carotene on the outer layer of the wall materials might have resulted in a rapid release in SGJ after 30 min. The non-significant change in beta-carotene release from SSB after 60 min of SGJ illustrates the starch’s ability to keep beta-carotene molecules inside its cavity. When the enclosed systems are exposed to simulated colonic circumstances, the bioactive carrier system gradually degrades with increasing duration, i.e., 30 and 60 min, and the release of beta carotene increases dramatically after 120 min. After 120 min, the SSB had a maximum release of 8.02 g/mL. The increased surface area of the nano-encapsulating systems at greater pH levels and in the presence of the bile salts may have impaired their capacity to release beta-carotene quickly [33]. The higher the release of beta carotene in SIJ, the greater will be the health beneficial effects on the host. A similar gradual release pattern was found from the microencapsules of caffeine loaded in polysaccharide-based delivery systems by Noor et al., 2018 [34], In another study, it has been reported that starch nanoparticles have better cell permeability and bio-accessibility and also protected catechin during the digestion process [30]. (Fig. 3).

### 3.7 Anti-oxidant potential of nanocapsules

When DPPH gets in contact with a sample that donates protons, the stable free radical quickly decreases, revealing the sample’s anti-oxidant potential. The effects of radical scavenging are assessed by evaluating absorbance at 517 nm and observing a shift in color from violet to pale yellow [35]. The DPPH free radical scavenging assay was done to evaluate antioxidant capability of formulated nanocapsules, the findings are displayed in Table 2. FSB nanocomplex has shown stronger antioxidant activity than SSB and PSB nanocomplex which might be due to higher encapsulation efficiency of FSB. As radical scavenging activity is concentration-dependent the encapsulated bioactive boosts the scavenging activity of nanoparticles. The strong scavenging activity is attributed to the presence of bioactives (phenolics, carotenoids, tocols, etc.) that allow the nanoparticles to act as antioxidants [36]. It is further increased by the breakdown of hydrogen bonds of starch during ultrasonication. The breaking of bonds may reveal more hydroxyl groups in modified polysaccharides, allowing more scavenging of DPPH radicals [37].

**Table 2:** DPPH, Metal chelating activity and reducing power of nanocapsules.

Sample	DPPH (%)	Metal chelating activity (%)	Reducing power (%)
SSB	69.02 ± 0.02^b^	74.03 ± 0.03^b^	63.09 ± 0.07^b^
FSB	70.21 ± 0.01^c^	77.89 ± 0.23^c^	56.98 ± 0.21^c^
PSB	65.06 ± 0.12^a^	60.34 ± 0.04^a^	49.03 ± 0.32^a^

Where SSB, FSB, and PSB represent Beta carotene loaded starch nanocapsules prepared from sorghum, foxtail millet, and pearl millet starch respectively. The presence of distinct small superscript letters (a, b & c) on the mean data alongside the standard deviation (±) within the identical column indicates significant differences (*p* < 0.05) among them.

The reducing power of all samples is shown in Table 2. The reducing power is contingent on the material’s ability to provide hydrogen molecules to the ferric ion (Fe_3_^+^), reducing it to the ferrous ion (Fe_2_^+^) in this manner. After the process, the ferric ferrous complex exhibits absorption at 700 nm. The findings showed that there was a statistically significant variance (*p* < 0.05) among all samples. Since reducing power and antioxidant activity are strongly correlated, SSB showed the best potential for reducing power. It demonstrates that it is highly capable of halting the free radical chain reaction and indicates its antioxidant abilities.

The chelation of metals that promote oxidation is one of the most significant mechanisms of secondary antioxidant effects. Certain metals, including iron and other transition metals like copper, chromium, cobalt, vanadium, cadmium, and nickel which operate as catalysts for free radical processes that encourage oxidation. Chelation of metals by specific chemicals lowers their pro-oxidant action by lowering their redox potentials and the metal’s oxidized state. [38]. In comparison to PSB and SSB the chelation activity was shown to be greater in FSB as illustrated in Table 2. The strong chelation activity of FSB might be attributed to the presence of higher percent of beta carotene present in the core of the nanocapsule as indicated in Table 1. Beta carotene with its strong ferrous ion chelating activity are regarded to be desirable for inclusion in meals because ferrous ions effectively produce oxidative stresses in the food system.

## 4 Conclusions

Millet nano starch was prepared using acid hydrolysis method and beta carotene was then successfully encapsulated in nano starch using ultrasonication method. Millet starch nanoparticles positively shielded beta carotene from the harsh environment and transported it at its utilization site in the gut. The bioactivity of beta carotene was well retained in the nano starch matrix. Millet nano starch’s desirable *in-vitro* release of beta carotene under various gastrointestinal conditions offers its possible use to serve as a nano-vehicle for target delivery and the discharge of bioactive at specific sites for the fabrication of future nutritious food products and use in the field of medicine. To optimize the potential medicinal properties of encapsulated beta carotene, more investigation must be conducted to figure out how to utilize it in various food platforms.

## CRediT authorship contribution statement

**Mehak Nazir:** Writing – original draft, Investigation. **Faiza Jhan:** Conceptualization, Formal analysis, Writing – review & editing. **Asir Gani:** Formal analysis. **Adil Gani:** Supervision.

## Funding

Dr. Adil Gani and Dr. Faiza Jhan are thankful to department of science and technology (DST), Government of India for the award of WISE-post-doctoral fellowship (DST/WISE-PDF/LS-117/2023).

## Declaration of competing interest

The authors declare that they have no known competing financial interests or personal relationships that could have appeared to influence the work reported in this paper.
